# Can abscopal effects of local radiotherapy be predicted by modeling T cell trafficking?

**DOI:** 10.1186/s40425-016-0133-1

**Published:** 2016-05-17

**Authors:** Sandra Demaria, Silvia C. Formenti

**Affiliations:** Department of Radiation Oncology, Weill Cornell Medicine, 1300 York Ave, Box 169, New York, NY 10065 USA

**Keywords:** Abscopal effect, T cell trafficking, Mathematical model

## Abstract

The abscopal effect of radiation describes tumor regression in metastases outside of the field upon treatment of one site, and is mediated by radiation-induced anti-tumor T cells. The ability of radiation to generate an in situ tumor vaccine and improve responses to immunotherapy is under intense investigation in the clinic. Preclinical and clinical evidence shows that multiple factors regulate radiation interaction with the immune system within and outside of the irradiated tumor. Poleszczuk and colleagues developed a mathematical model of T cell trafficking between metastases, and in a recent publication propose that the specific metastatic site irradiated determines the ability of T cells to traffic to other metastases and mediate abscopal responses and should dictate clinical decision making [Poleszczuk et al. Cancer Res 76:1009-18, 2016]. Here we critically discuss this model in light of the currently available information about abscopal responses in mice and patients. Caution in relying upon overly simplified models, before validation in real patients, is recommended.

## Text

The term “abscopal effect” has been used for over sixty years to describe the rare occurrence of tumor regression in metastases away from the irradiated target lesion, hence “ab scopus” which in Latin means outside of the target [1]. Twelve years ago we linked the abscopal effect to immunity, when we demonstrated an out of field response in a syngeneic murine model of mammary carcinoma treated by localized radiation and FMS-like tyrosine kinase 3 ligand (Flt-3L), that was abrogated in similar experiments performed in nude mice [2]. In recent years, the term has gained popularity due to reports of abscopal responses in melanoma patients treated with local radiotherapy while they were progressing during treatment with anti-CTLA-4 antibody [3]. Evidence that the abscopal effect is mediated by radiation-induced anti-tumor T cells, and can be induced in mice and patients by combining local radiation with growth factors for dendritic cells, Toll Like Receptor (TLR) agonists or immune checkpoint inhibitors, has opened a new field of investigation [2, 4–7]. Close to hundred trials are ongoing, to test the ability of local radiotherapy to induce an in situ tumor vaccine and enhance systemic responses to immune checkpoint inhibitors in cancer patients, or to test the combination of radiation with a variety of other immune modulators (clinicaltrials.gov).

Several questions remain unanswered about the optimal use of radiation to convert the irradiated tumor into an in situ vaccine and achieve abscopal responses, including the dose, schedule and technique of radiation, the sequencing with different immune modulators, and the susceptibility of different tumor types and carriers [8]. An intriguing question is whether the location of the tumor that is irradiated contributes to the likelihood of inducing abscopal responses. In a recently published article Jan Poleszczuk and colleagues have generated a mathematical model to predict the answer to this question [9]. To build the model they hypothesized that abscopal responses can only be achieved if T cells activated at one tumor site reach each of the other metastases in sufficient numbers. In addition, they made the assumption that trafficking of the activated T cells from the site of activation (the irradiated tumor) to a given organ containing metastases will be determined by the physiologic blood flow to that organ, and by the initial imprinting of the T cells by tumor antigen-presenting dendritic cells, which confer tissue-tropism to the primed T cells. Using these parameters they calculated the “immunogenicity index” of metastatic sites in virtual patients. They propose that their model can be applied to identify treatment targets with the highest likelihood of inducing abscopal effects, which are patient-specific and not otherwise intuitively predictable.

The limitations of the model stem from its virtual foundations. Instead of generating an annotated registry of abscopal reponders [3, 7] and interrogating such a database to design the model, the authors have generated an “in silico” set of 40 virtual “patients”, to model their theory of T cell trafficking and homing. The limitations are more evident when the model is confronted with the abscopal effects of radiotherapy in real patients: clinical out of the field responses occur over a period of months, suggesting that the speed or efficiency of initial trafficking from the irradiated tumor and/or tumor-draining lymph nodes where the priming occurred may not be a critical factor [3, 6, 7]. Rather, immunogenic cell death induced by radiation is a gradual process, evolving beyond the completion of radiation treatment. Factors that favor or hinder local proliferation and expansion of the anti-tumor T cells at a given metastatic site are more likely to be critical determinants of the abscopal response. For instance the common expression of antigen(s) or neoantigens between the irradiated metastasis and other tumor sites may be key to an abscopal response. In our experience abscopal reponses in patients were metachronous, with each metastasis regressing at different times [6, 7]. Thus, rejection of a tumor at one abscopal site can prime new T cells or expand the T cell originally primed at the irradiated site, generating a T cell response that is broader not only in number of effector T cells, but also in their antigenic specificity and tissue tropism.

Another fundamental parameter that is missing in the model relates to the effect of the specific radiotherapy used to trigger an abscopal response. Both the dose and fractionation regimen as well as the target volume chosen directly impact adequacy of naïve T cells indispensible for successful immunization. In a model of a classical 8 cm diameter target, treated with the classical 30 fractions of 2 Gy each of standard radiotherapy, 99 % of the circulating blood cells were likely to receive >0.5 Gy, a dose sufficient to markedly reduce the number of available naïve T cells at the time of cross presentation [10].

Overall, it is possible that the specific location of the irradiated metastasis may influence the chances for generation of an in situ vaccine by radiation. However, we reckon that the local immune microenvironment, and especially the availability of dendritic cells, which cross-present the antigens released by radiation, and of sufficient naïve T cells at the time of cross presentation determines the magnitude of the anti-tumor immune responses primed and likely trumps blood flow [2, 6]. Radiation not only induces an immunogenic death of the cancer cells, but enhances pro-inflammatory chemokines and vascular adhesion molecules, as well as major histocompatibility class I (MHC-I), natural-killer group 2, member D (NKG2D) ligands and Fas/CD95 expression on the cancer cells that survive, effectively converting the irradiated tumor into an immunogenic hub, where cycles of antigen release and cross-presentation can occur over time [8]. The degree to which each of these pro-immunogenic processes will take place depends on the balance of the pre-existing immunosuppressive factors with the activating and suppressive signals generated by radiation. In a metastatic setting, radiation alone seldom shifts the balance towards immune activation, as demonstrated by the rarity of abscopal effects; however when combined with immune modulators abscopal effects occur in approximately a third of patients [5, 6]. In this context, the size of the tumor chosen for radiation may play a complex role: while presumably a larger tumor could release more antigens, both quantitatively and qualitatively (e.g., a larger variety of neoantigens), it is also likely to contain more areas of hypoxia, which are both, radioresistant and highly immunosuppressive. Additionally, radiation targeting of larger tissue volumes, in deeper sites, exposes more circulating naïve T cells to cytocidal doses, with consequent lymphopenia [10]. Thus, overlapping sets of variables that are likely to influence clinical outcome need to be reckoned with to develop a clinically meaningful model.

In conclusion, more information about the determinants of the abscopal effect is needed to guide the design of clinical trials testing local radiation with the rapidly growing number of immunotherapy agents. Mathematical modeling is a welcome addition to the cadre of experimental approaches used to interrogate the mechanisms of the abscopal effect. However, the potential impact of a model is dictated by its ability to comprehensively address the contribution of multiple interrelated factors in achieving the outcome studied, and by rigorous validation against any available clinical information. An oversimplified model such as the one described by Jan Poleszczuk and colleagues [9] not only has limited usefulness, but also risks misleading the design of clinical studies.
